# The impact of memory effect on space fractional strong quantum couplers with tunable decay behavior and its numerical simulation

**DOI:** 10.1038/s41598-021-89701-7

**Published:** 2021-05-13

**Authors:** Ahmed S. Hendy, Mahmoud A. Zaky, Ramy M. Hafez, Rob H. De Staelen

**Affiliations:** 1 Department of Computational Mathematics and Computer Science, Institute of Natural Sciences and Mathematics, Ural Federal University, 19 Mira St., Yekaterinburg, Russia 620002; 2 Department of Mathematics, Faculty of Science, Benha University, Benha, 13511 Egypt; 3 Department of Applied Mathematics, Physics Division, National Research Centre, Dokki Cairo, 12622 Egypt; 4 Faculty of Education, Matrouh University, Matrouh, Egypt; 5 Dean’s Office of the Faculty of Medicine and Health Sciences, Ghent University, C. Heymanslaan 10, Ghent, 9000 Belgium; 6 Ghent University Hospital, C. Heymanslaan 10, Ghent, 9000 Belgium

**Keywords:** Computational science, Applied mathematics

## Abstract

The nontrivial behavior of wave packets in the space fractional coupled nonlinear Schrödinger equation has received considerable theoretical attention. The difficulty comes from the fact that the Riesz fractional derivative is inherently a prehistorical operator. In contrast, nonlinear Schrödinger equation with both time and space nonlocal operators, which is the cornerstone in the modeling of a new type of fractional quantum couplers, is still in high demand of attention. This paper is devoted to numerically study the propagation of solitons through a new type of quantum couplers which can be called time-space fractional quantum couplers. The numerical methodology is based on the finite-difference/Galerkin Legendre spectral method with an easy to implement numerical algorithm. The time-fractional derivative is considered to describe the decay behavior and the nonlocal memory of the model. We conduct numerical simulations to observe the performance of the tunable decay and the sharpness behavior of the time-space fractional strongly coupled nonlinear Schrödinger model as well as the performance of the numerical algorithm. Numerical simulations show that the time and space fractional-order operators control the decay behavior or the memory and the sharpness of the interface and undergo a seamless transition of the fractional-order parameters.

## Introduction

Recently, optical solitons have arisen in various optical systems, which have many applications^1–3^. There exist different classifications of optical solitons. Temporal solitons and spatial solitons are the two main classifications. The combination of these two classifications forms spatiotemporal solitons. This kind of solitons, which are named light bullets, can be formed in the support of nonlinear effect in which a compensation of light beams spatial diffraction (broadening) and laser pulses temporal dispersion (spreading) occurs^1,3,4^. Information technology, like the internet and other telecommunication networks, is based on optical fiber technology. This technology is used widely in biomedical and biological studies. In the fabrication process of conventional optical fibers, it is noticed that some optical properties, apart from the transmission of light, can’t be easily modified and preserved. Routing, switching, and buffering of information are some of these optical properties. Because of that, the efforts of researchers in recent years are devoted to using dual-core fibers to develop and enhance fiber optic sensing techniques^5,6^. The ability of the new dual-core fibers to perform the same functions more precisely is proved in^7^ by applying a minuscule amount of mechanical pressure. In other words, an improvement is reported in optical fiber-based technology by the mechanism of two cores fiber fabrication which is based on moving the cores close enough to each other.

More recently, a nanomechanical optical fiber fabrication and demonstration are reported^7^. The main feature of that optical fiber is its core which has a great role in controlling the nanometer-scale mechanical movements side by side to the transmission of light. The methodology of the fabricated fibers which have two movable cores is based on making their cores more close to each other to have the opportunity to act as a directional coupler. Armed by that, the light can make a jump between the two cores which are separated only by a few nanometers. Some recent applications are based on that new technology. An example of these applications is optical buffering which can enhance processing, routing, and switching properties over long distances. Owing to potential applications in optical communication, propagation of solitons through optical fibers in most optical communication systems became a wide area of research. The integrable nonlinear Schrödinger’s equation (NLSE)^8,9^ is used to model these dynamics. A more generalized form^10^, known as the generalized nonlinear Schrödinger’s equation (GNLSE), is given by

1 $$\begin{aligned} \textit{i}u_t+\frac{1}{2}u_{xx}+{\mathscr {F}}\left( |u|^2\right) u=0, \end{aligned}$$

where ${\mathscr {F}}$ is a real-valued algebraic function which can be considered as the refractive index of the fibers. Earlier work^10^ extended the GNLSE to the couplers case. Fast switching and signal coupling in optical communication links can be allowed by optical nonlinear couplers. For twin-core couplers, wave propagation at relatively high field intensities is described by coupled nonlinear equations. In the dimensionless form, they are:

2 $$\begin{aligned}&\textit{i}u_t+\frac{1}{2}u_{xx}+{\mathscr {F}}\left( |u|^2\right) u={\mathscr {K}}\nu , \end{aligned}$$

3 $$\begin{aligned}&\textit{i}\nu _t+\frac{1}{2}\nu _{xx}+{\mathscr {F}}\left( |\nu |^2\right) \nu ={\mathscr {K}}u. \end{aligned}$$

The coupling coefficient between the cores of the fiber is represented by the constant ${\mathscr {K}}$. The system (2) and (3) has been derived and studied before in the context of Kerr law nonlinearity^11^. The dimensionless forms of the optical fields in the respective cores of the optical fibers are given by *u* and $\nu$. This system of equations is known as the generalized vector NLSE. Intensity dependent switches and devices for separating a compressed soliton from its broad ‘pedestal’ are examples of applications of (2) and (3). Its integrals of motion can be respectively given by energy (*E*), linear momentum (*M*) and the Hamiltonian (*H*) namely:

4 $$\begin{aligned} E= & {} \int _{-\infty }^{\infty }(|u|^2+|\nu |^2)dx, \end{aligned}$$

5 $$\begin{aligned} M= & {} \frac{\textit{i}}{2}\int _{-\infty }^{\infty }\left[ (u u_x^{*}-u^*u_x)+(\nu \nu _x^{*}-\nu ^*\nu _x)\right] dx, \end{aligned}$$

6 $$\begin{aligned} H= & {} \int _{-\infty }^{\infty }\left[ \frac{1}{2}\left( |u_x|^2+|\nu _x|^2\right) -\mathrm {F}\left( |u|^2\right) -\mathrm {F}\left( |\nu |^2\right) -k\left( u \nu ^*+\nu u^*\right) \right] dx, \end{aligned}$$

where we define $\mathrm {F}(I)=\int _{0}^{I}{\mathscr {F}}(\zeta )d\zeta ,$ and the intensity *I* is given by $I=|u|^2$ or $I=|\nu |^2$ depending on the core. The energy *E* is commonly known as the wave energy and in the context of fiber optics it is known as the wave power.

The theory which used to discuss quantum phenomena in fractal environments is called fractional quantum mechanics. The similarity between the classical diffusion equation and the Schrödinger equation motivated the generalization of the nonlinear Schrödinger equation in the light of non-Brownian motion in a path integral formulation. This generalization leads to the space-fractional, the time-fractional, and the time-space-fractional Schrödinger equations (FSEs). The Riesz space FSE has been introduced by Laskin in quantum physics by replacing the Brownian paths in the Feynman path integrals by Lévy flights^12,13^. Similar to the conventional Schrödinger equation, the Riesz space FSE satisfies the Markovian evolution law. Stickler^14^ discussed the Lévy crystal in a condensed matter environment as a possible realization of the space-fractional quantum mechanics by introducing a tight binding infinite range chain. Longhi^15^ discussed an optical realization of the space FSE based on transverse light dynamics in a cavity by exploiting the Fourier optics properties. Other physical applications of the Riesz space FSE have been discussed by Guo and Xu^16^ and its solution for a free particle and an infinite square potential well have also been introduced. The existence and uniqueness of the solution to the space FSE have been investigated by Guo et al.^17^ using the energy method. Moreover, the existence and uniqueness of the solution to systems of the space FSEs have been proved by Hu et al.^18^ using the Faedo-Galerkin method. Cho et al.^19^ studied the low regularity well-posedness of the space FSE with cubic nonlinearity in periodic and non periodic settings. Following Laskin and similar to deriving the time-fractional diffusion equation by considering non-Markovian evolution^20^, Naber^21^ used the Caputo temporal fractional derivative^22^ as a generalization of the integer-order derivative in the conventional Schrödinger equation to study non-Markovian evolution in quantum mechanics and constructed the temporal FSE. More recently, Dong and Xu^23^, and Wang and Xu^24^ combined Laskin’s work with Naber’s work to construct space-time FSEs. A detailed derivation and numerical simulation of coupled system of nonlinear Schrödinger equations for pulses of polarized electromagnetic waves in cylindrical fibers was investigated^25^. Ghalandari and Solaimani^26^ investigated the numerical treatment of the fractional Young double-slit experiment with incident Gaussian wavepackets using a split step Fourier method. Liangwei Zeng and Jianhua Zeng^27^ proposed a coupled system of space FSEs considering two arrays of quantum waveguides. They considered the following system with the wave function $u,\nu$:

7 $$\begin{aligned} \begin{aligned} \textit{i}\hbar \frac{\partial u}{\partial \tau }&=D_{\alpha }\left( -\hbar ^2\frac{\partial ^2}{\partial x^2}\right) ^{\frac{\alpha }{2}}u -G_1{\mathscr {F}}\left( |u|^{2}\right) u-{\mathscr {K}} \nu ,\\ \textit{i}\hbar \frac{\partial \nu }{\partial \tau }&=D_{\alpha }\left( -\hbar ^2\frac{\partial ^2}{\partial x^2}\right) ^{\frac{\alpha }{2}}\nu -G_2{\mathscr {F}}\left( |\nu |^{2}\right) \nu -{\mathscr {K}} u, \end{aligned} \end{aligned}$$

where $\hbar$ represents the Planck constant, the differentiation order parameter $\alpha$ represents the Lévy index, $\tau$ is the time, and *K* is the linear coupling parameter. The parameter $D_\alpha = 1/m$ is constant with *m* denoting the mass of the atom. When ${\mathscr {F}}(S)=S$, then the nonlinear term is of Kerr style and $G_{1,2}>0$ are the Kerr coefficients. These coefficients can make a characterization of atoms collisions strengths in Bose–Einstein condensates. A kind of normalization can be applied on (7) by defining new variables $t=\tau /\tau _0,$ $\psi =\sqrt{h^{-\alpha }}u,$ $\phi =\sqrt{h^{-\alpha }}\nu ,$ $k={\mathscr {K}}/\left( D_{\alpha }\hbar ^{\alpha }\right),$ $g_{1,2}=G_{1,2}/D_{\alpha },$ and $\tau _0=\hbar ^{1-\alpha }/(D_{\alpha }).$ The fractional Laplacian is defined as $\left( -\nabla ^2\right) ^{\alpha /2}=\left( -\frac{\partial ^2}{\partial x^2}\right) ^{\alpha /2}.$ Accordingly, the system (7) should have the following dimensionless form:

8 $$\begin{aligned}  \textit{i}\frac{\partial \psi }{\partial t}&=\left( -\nabla ^2\right) ^{\frac{\alpha }{2}}\psi -g_1~{\mathscr {F}}\left( |\psi |^{2}\right) \psi -k \phi ,\\ \textit{i}\frac{\partial \phi }{\partial t}&=\left( -\nabla ^2\right) ^{\frac{\alpha }{2}}\phi -g_2~{\mathscr {F}}\left( |\phi |^{2}\right) \phi -k \psi . \end{aligned} $$

Based on (8), two arrays of quantum waveguides composition represent a new kind of space-fractional quantum couplers was considered in^27^. It is called space-fractional quantum couplers^27^. This can be understood in the sense of two branches polariton condensates in solid state physics^28^. The wave packets in such nonlinear fractional systems are modeled by the coupled system of nonlinear fractional Schrödinger equations with linear coupling. The new version of the space-time fractional Schrödinger equation derived by Laskin^29^ contains two scale dimensional parameters. One of them is a time-fractional generalization of the famous Planck’s constant. The other one can be explained as a time-fractional generalization of the scale parameter emerging in fractional quantum mechanics. Then the dimensionless physical model that can effectively describe the time-fractional quantum coupling is based on a set of strongly NLFSEs as follows,

9 $$\begin{aligned} \left\{ \begin{array}{l} i\frac{{\partial ^\beta \psi }}{{\partial t^\beta }} - \varepsilon \left( { - \Delta } \right) ^{\alpha /2} \psi + \left[ {k_1 \left| \psi \right| ^2 + \left( {k_1 + 2k_2 } \right) \left| \phi \right| ^2 } \right] \psi + \xi \phi = 0,\quad x \in \Lambda ,\ t \in J,\\ \\ i\frac{{\partial ^\beta \phi }}{{\partial t^\beta }} - \varepsilon \left( { - \Delta } \right) ^{\alpha /2} \phi + \left[ {k_1 \left| \phi \right| ^2 + \left( {k_1 + 2k_2 } \right) \left| \psi \right| ^2 } \right] \phi + \xi \psi = 0,\quad x \in \Lambda ,\ t \in J,\\ \\ \psi (x,0) = \psi _0 ,\phi (x,0) = \phi _0,\quad x \in \Lambda ,\\ \\ \psi (x , t)=\phi (x , t) =0, \qquad x\in \partial \Lambda ,\ t \in J,\\ \end{array} \right. \end{aligned}$$

where $$\Lambda =(a , b) \subset {\mathbb {R}}$$ and $$ J=(0,T] \subset {\mathbb {R}}$$ are the time and the space domains, $$\partial \Lambda $$ is the boundary of $$\Lambda 
$$, the parameter $$\varepsilon >0$$ is the group velocity dispersion, $$\gamma $$ denotes the normalized birefringence constant, $$\psi $$ and $$ \phi $$ are complex functions defined in $$\Lambda \times J$$, $$\psi _{\mathrm {0}}(x)$$ and $$\phi _{\mathrm {0}}(x)$$ are known sufficiently smooth functions. Of particular concern for the present work is the linear coupling parameter $$\xi $$. It accounts for effects that result from the twisting and elliptic deformation of the fiber. The term proportional to $$k_1$$ explains the self-focusing of a signal for pulses in birefringent media whereas $$k_1+2k_2$$ represents the cross phase modulation describing the integrability of the nonlinear system. The operator $$\frac{\partial ^\beta }{\partial t ^\beta }\ (0<\beta <1)$$ is the Caputo fractional derivative, and $$(-\Delta )^{\alpha /2}$$ is the fractional order Laplacian operator of order $$1<\alpha \le 2$$ defined in Riesz form $$\frac{\partial ^\alpha }{\partial |x|^\alpha }\ (1<\alpha <2) $$. We refer to seminal works^30^ for more details on fractional order differential operators. We here numerically solve model (9) and provide an easy to implement algorithm in the next section.

In this paper, we numerically investigate the nontrivial behavior of wave packets in the time-space fractional model (9) in one space dimension. In this model, we choose the form of the fractional Laplacian to be the Riesz fractional operator. The time-fractional derivative is considered to describe the decay behavior and the non-local memory of the model. The numerical methodology is based on the finite difference/Galerkin–Legendre spectral method with an easy to implement numerical algorithm. We conduct numerical simulations to demonstrate the performance of the tunable decay and the sharpness behavior of the time-space fractional strongly coupled nonlinear Schrödinger model as well as the the performance of the numerical algorithm. Numerical simulations show that the time and space fractional order operators control the decay behavior or the memory and the sharpness of the interface and undergo a seamless transition of the fractional order parameters. Of particular interest for this work also is the linear coupling parameter $\xi$. We test the effect of this parameter on the collision of solitary waves.

## The spectral scheme

Here, the discretization of problem (9) is done by using the high order *L*2-$1_\sigma$ approximation difference formula for the Caputo time fractional operator next to the spectral Legendre–Galerkin scheme for the Riesz spatial-fractional operator. The time and space fractional Schrödinger equalion and its coupled system are fully analyzed numerically in^31,32^. The proposed numerical approaches there are designed by the use of L1 difference scheme for temporal approximations and Galerkin spectral scheme for spatial fractional order operators. Alternatively, Fourier spectral method can be used effectively for Riesz space fractional partial differential equations. For example, a fast and accurate method for numerical solutions of space fractional reaction-diffusion equations is proposed in^33^ based on an exponential integrator scheme in time and the Fourier spectral method in space. By the Fourier spectral techniques and advance the resulting equation in time with both Strang splitting and exponential time-differencing methods, the dynamics of the time-dependent Riesz space fractional nonlinear Schrödinger equation in the presence of the harmonic potential have been considered in^34^. A detailed implementation of the constructed high accuracy algorithm for (9) will be given below.

### Formalism

We partition the temporal domain *J* by $t_s=s \tau ,$ $s=0,\ 1, \ldots , M$ where $\tau = T/M$. Denote $t_{s+\sigma } = (s+\sigma )\tau =\sigma t_{s+1}+(1-\sigma )t_{s},$ for $s = 0,\ 1, \ldots , M-1$. Let $\Psi ^{s+\sigma } =\Psi ^{s+\sigma }(\cdot )= \Psi (\cdot ,t_{s+\sigma })$.

#### Definition 1

Let $0< \beta < 1$ and $\sigma = 1 - \frac{\beta }{2}$. Define

10 $$\begin{aligned} a _s ^{(\beta , \sigma )}= & {} \left\{ \begin{array}{ll} \sigma ^{1 - \beta } , &{} s = 0, \\ (s + \sigma ) ^{1 - \beta } - (s - \sigma +1) ^{1 - \beta }, &{} s \ge 1, \end{array} \right. \end{aligned}$$

11 $$\begin{aligned} b _s ^{(\beta , \sigma )}= & {} \frac{1}{2 - \beta } \left[ ( \sigma +s) ^{2 - \beta } - (s - \sigma +1) ^{2 - \beta } \right] - \frac{1}{2} \left[ (s + \sigma ) ^{1 - \beta } + (s - \sigma +1) ^{1 - \beta } \right] , \quad s \ge 1, \end{aligned}$$

and

12 $$\begin{aligned} C _s ^{(j,\beta , \sigma )} = \left\{ \begin{array}{ll} a _0 ^{(\beta , \sigma )}, &{} s = j =0, \\ a _0 ^{(\beta , \sigma )} + b _1 ^{(\beta , \sigma )}, &{} s = 0,\ j \ge 1, \\ a _s ^{(\beta , \sigma )} + b _{s + 1} ^{(\beta , \sigma )} - b _s ^{(\beta , \sigma )}, &{} 1 \le s \le j-1, \\ a _j ^{(\beta , \sigma )} - b _j ^{(\beta , \sigma )}, &{} 1 \le s = j. \end{array} \right. \end{aligned}$$

#### Lemma 2.1

(Alikhanov difference formula^35^) *The high order Alikhanov* $L _2$-$1 _\sigma$ *difference formula under the assumption* $\Psi (t) \in C^{3}[0,t_{j+1}]$, $0\le j \le M-1$, *formulated as*

13 $$\begin{aligned} {} _0 D _{\tau } ^\beta \Psi ^{j + \sigma } = \frac{\tau ^{- \beta }}{\Gamma (2 - \beta )} \sum _{r = 0} ^j C _{j - r} ^{(j,\beta , \sigma )} \delta _{t}\Psi ^{r}+ {\mathscr {O}} (\tau ^{3 - \beta }),\qquad 0< \beta < 1 , \end{aligned}$$

*where* $\delta _{t}\Psi ^{r}= \Psi ^{r + 1} - \Psi ^r$, *can be rewritten as*

14 $$\begin{aligned} {} _0 D _{\tau } ^\beta \Psi ^{j + \sigma } = \frac{\tau ^{- \beta }}{\Gamma (2 - \beta )} \sum _{r = 0} ^j d _{ r} ^{(j,\beta , \sigma )} \Psi ^{r}+ {\mathscr {O}} (\tau ^{3 - \beta }), \end{aligned}$$

*where* $$d _{1} ^{(0,\beta , \sigma )}=-d _{0} ^{(0,\beta , \sigma )}=\sigma ^{1-\beta }$$ $$\forall \,j=0$$, *and* $$\forall \,j\ge 1,$$

15 $$\begin{aligned} d _s ^{(j,\beta , \sigma )} = \left\{ \begin{array}{ll} -C _j ^{(j,\beta , \sigma )} , &{} s = 0, \\ C_{j-s+1} ^{(j,\beta , \sigma )}-C_{j-s} ^{(j,\beta , \sigma )} , &{} 1 \le s \le j, \\ C_0 ^{(j,\beta , \sigma )} , &{} s = j+1. \end{array} \right. \end{aligned}$$

#### Definition 2

Let $j \in {\mathbb {Z}} _{[0 , M-1]}$, the Alikhanov *L*2-$1 _\sigma$ *difference formula* at the node $t _{j+\sigma }$ is defined as

16 $$\begin{aligned} {} _0 D _\tau ^\beta \Psi ^{j + \sigma } = \frac{\tau ^{- \beta }}{\Gamma (2 - \beta )} \sum _{r = 0} ^{j+1} d_{r}^{(j,\beta , \sigma )} \Psi ^{r},\qquad 0< \beta < 1. \end{aligned}$$

The next identity holds directly by Taylor’s theorem.

#### Lemma 2.2

*The following identity holds:*

17 $$\begin{aligned} \Psi (\cdot , t _{j + \sigma })&= \sigma \Psi (\cdot , t _{j + 1}) + (1 - \sigma ) \Psi (\cdot , t _j) + {\mathscr {O}} (\tau ^2). \end{aligned}$$

Starting from the *L*2-$1 _\sigma$ formula (16) for the discretization of the time Caputo fractional derivative, we obtain that

18 $$\begin{aligned} \left\{ \begin{array}{l} i\,{}_0D_\tau ^\beta \psi ^{j + \sigma } - \varepsilon \left( { - \Delta } \right) ^{\alpha /2} \psi ^{j + \sigma } + \left[ {k_1 \left| {\psi ^{j + \sigma } } \right| ^2 + \left( {k_1 + 2k_2 } \right) \left| {\phi ^{j + \sigma } } \right| ^2 } \right] \psi + \gamma \psi ^{j + \sigma } + \xi \phi ^{j + \sigma } = 0,\\ \\ i\,{}_0D_\tau ^\beta \phi ^{j + \sigma } - \varepsilon \left( { - \Delta } \right) ^{\alpha /2} \phi ^{j + \sigma } + \left[ {k_1 \left| {\phi ^{j + \sigma } } \right| ^2 + \left( {k_1 + 2k_2 } \right) \left| {\psi ^{j + \sigma } } \right| ^2 } \right] \phi + \gamma \phi ^{j + \sigma } + \xi \psi ^{j + \sigma } = 0,\\ \\ \end{array} \right. \end{aligned}$$

Making use of Lemma 2.1 and Lemma 2.2, this scheme is of second order accuracy in time. Let us introduce the following parameters

$$\begin{aligned} \xi _{j}^{(\beta ,\sigma )} = \left( {\frac{{d_{j + 1}^{(j,\beta ,\sigma )} }}{{\tau ^\beta \Gamma (2 - \beta )}} } \right) ^{-1}, \qquad {{\tilde{d}}}_{i}^{(j,\beta ,\sigma )} = \frac{{\xi _{j}^{(\beta ,\sigma )} d_i^{(j,\beta ,\sigma )} }}{{\tau ^\beta \Gamma (2 - \beta )}}, \ \ 0 \le i \le j . \end{aligned}$$

The semi-scheme (18) has the following equivalent form:

19 $$ \begin{aligned} \left\{ \begin{array}{l} \begin{array}{l} i\, \psi ^{j + 1} - \varepsilon \sigma \xi _j^{\left( {\beta ,\sigma ,\gamma } \right) } \left( { - \Delta } \right) ^{\alpha /2} \psi ^{j + 1} = \varepsilon (1 - \sigma )\xi _j^{\left( {\beta ,\sigma ,\gamma } \right) } \left( { - \Delta } \right) ^{\alpha /2} \psi ^j - \sum \limits _{q = 0}^j {d_q^{\left( {j,\beta ,\sigma } \right) } \psi ^q } \\\,\, \quad\quad - \sigma \xi _j^{\left( {\beta ,\sigma ,\gamma } \right) } \left[ {k_1 \left| {\psi ^{j + 1} } \right| ^2 + \left( {k_1 + 2k_2 } \right) \left| {\phi ^{j + 1} } \right| ^2 } \right] \psi ^{j + 1} - \sigma \xi _j^{\left( {\beta ,\sigma ,\gamma } \right) } \xi \phi ^{j + 1} \\  \,\,\quad\quad - (1 - \sigma )\xi _j^{\left( {\beta ,\sigma ,\gamma } \right) } \left[ {k_1 \left| {\psi ^j } \right| ^2 + \left( {k_1 + 2k_2 } \right) \left| {\phi ^j } \right| ^2 } \right] \psi ^j -(1 - \sigma )\xi _j^{\left( {\beta ,\sigma ,\gamma } \right) } \xi \phi ^j, \\ \end{array}\\ \\ \begin{array}{l} i\,\phi ^{j + 1} - \varepsilon \sigma \xi _j^{\left( {\beta ,\sigma ,\gamma } \right) } \left( { - \Delta } \right) ^{\alpha /2} \phi ^{j + 1} = \varepsilon (1 - \sigma )\xi _j^{\left( {\beta ,\sigma ,\gamma } \right) } \left( { - \Delta } \right) ^{\alpha /2} \phi ^j - \sum \limits _{q = 0}^j {d_q^{\left( {j,\beta ,\sigma } \right) } \phi ^q } \\ \,\,\quad \quad -\sigma \xi _j^{\left( {\beta ,\sigma ,\gamma } \right) } \left[ {k_1 \left| {\phi ^{j + 1} } \right| ^2 + \left( {k_1 + 2k_2 } \right) \left| {\psi ^{j + 1} } \right| ^2 } \right] \phi ^{j + 1} - \sigma \xi _j^{\left( {\beta ,\sigma ,\gamma } \right) } \xi \psi ^{j + 1} \\ \,\,\quad\quad - (1 - \sigma )\xi _j^{\left( {\beta ,\sigma ,\gamma } \right) } \left[ {k_1 \left| {\phi ^j } \right| ^2 + \left( {k_1 + 2k_2 } \right) \left| {\psi ^j } \right| ^2 } \right] \phi ^j - (1 - \sigma )\xi _j^{\left( {\beta ,\sigma ,\gamma } \right) } \xi \psi ^j \\ \end{array}\\ \\ \end{array} \right. \end{aligned} $$

And so, the full discrete Alikhanov *L*2-$1 _\sigma$ Galerkin spectral scheme for (18) is to get $\psi _N^{j+1},\ \phi _N^{j+1} \in V_N^0,$ $j \ge 0,\ \forall \nu \in V_N^0$ such that

20 $$\begin{aligned} \left\{ \begin{array}{l} i\,\left( {\psi _N^{j + 1} ,v} \right) - \varepsilon \sigma \xi _j^{\left( {\beta ,\sigma ,\gamma } \right) } \left( { - \Delta } \right) ^{\alpha /2} \left( {\psi _N^{j + 1} ,v} \right) = \varepsilon (1 - \sigma )\xi _j^{\left( {\beta ,\sigma ,\gamma } \right) } \left( { - \Delta } \right) ^{\alpha /2} \psi _N^j - \sum \limits _{q = 0}^j {d_q^{\left( {j,\beta ,\sigma } \right) } \left( {\psi _N^q ,v} \right) } \\ \qquad \qquad - \sigma \xi _j^{\left( {\beta ,\sigma ,\gamma } \right) } \left( {I_N \left[ {k_1 \left| {\psi _N^{j + 1} } \right| ^2 + \left( {k_1 + 2k_2 } \right) \left| {\phi _N^{j + 1} } \right| ^2 } \right] \psi ^{j + 1} ,v} \right) - \sigma \xi _j^{\left( {\beta ,\sigma ,\gamma } \right) } \xi \left( {\phi _N^{j + 1} ,v} \right) \\ \qquad \qquad - (1 - \sigma )\xi _j^{\left( {\beta ,\sigma ,\gamma } \right) } \left( {I_N \left[ {k_1 \left| {\psi _N^j } \right| ^2 + \left( {k_1 + 2k_2 } \right) \left| {\phi _N^j } \right| ^2 } \right] \psi _N^j ,v} \right) - (1 - \sigma )\xi _j^{\left( {\beta ,\sigma ,\gamma } \right) } \xi \left( {\phi _N^j ,v} \right) ,\\ \\ i\,\left( {\phi _N^{j + 1} ,v} \right) - \varepsilon \sigma \xi _j^{\left( {\beta ,\sigma ,\gamma } \right) } \left( { - \Delta } \right) ^{\alpha /2} \left( {\phi _N^{j + 1} ,v} \right) = \varepsilon (1 - \sigma )\xi _j^{\left( {\beta ,\sigma ,\gamma } \right) } \left( { - \Delta } \right) ^{\alpha /2} \phi _N^j - \sum \limits _{q = 0}^j {d_q^{\left( {j,\beta ,\sigma } \right) } \left( {\phi _N^q ,v} \right) } \\ \qquad \qquad - \sigma \xi _j^{\left( {\beta ,\sigma ,\gamma } \right) } \left( {I_N \left[ {k_1 \left| {\phi _N^{j + 1} } \right| ^2 + \left( {k_1 + 2k_2 } \right) \left| {\psi _N^{j + 1} } \right| ^2 } \right] \phi ^{j + 1} ,v} \right) - \sigma \xi _j^{\left( {\beta ,\sigma ,\gamma } \right) } \xi \left( {\psi _N^{j + 1} ,v} \right) \\ \qquad \qquad - (1 - \sigma )\xi _j^{\left( {\beta ,\sigma ,\gamma } \right) } \left( {I_N \left[ {k_1 \left| {\phi _N^j } \right| ^2 + \left( {k_1 + 2k_2 } \right) \left| {\psi _N^j } \right| ^2 } \right] \phi _N^j ,v} \right) - (1 - \sigma )\xi _j^{\left( {\beta ,\sigma ,\gamma } \right) } \xi \left( {\psi _N^j ,v} \right) , \\ \\ \psi _N^0 = P_N\psi _0, \quad \phi _N^0 = P_N\phi _0,\\ \end{array} \right. \end{aligned}$$

where $P_N$ is the projection operator.

### Algorithmic implementation

Via the hypergeometric function, Jacobi polynomials can be represented^36^, for $\alpha ,\, \beta > -1$ and $x \in (-1,1)$:

21 $$\begin{aligned} J^{\alpha ,\beta }_{i}(x)=\frac{{(\alpha + 1)_i }}{{i!}}{}_2F_1 \left( { - i,\alpha + \beta + i + 1;\alpha + 1;\frac{{1 - x}}{2}} \right) ,\quad x \in (-1,1),\ i \in {\mathbb {N}}, \end{aligned} $$

where the notation $(\cdot )_i$ represents the symbol of Pochhammer. Armed by (20), we get the equivalent three-term recurrence relation

22 $$\begin{aligned} J^{\alpha ,\beta }_{0}(x)=&1,\\ J^{\alpha ,\beta }_{1}(x)=&\frac{1}{2}(\alpha +\beta +2)x+\frac{1}{2}(\alpha -\beta ), \\ J^{\alpha ,\beta }_{i+1}(x)=&\left( {\hat{a}}^{\alpha ,\beta }_i x-{\hat{b}}^{\alpha ,\beta }_i\right) J^{\alpha ,\beta }_{i}(x)-{\hat{c}}^{\alpha ,\beta }_i J^{\alpha ,\beta }_{i-1}(x),\quad i\ge 1, \end{aligned}$$

where

23 $$\begin{aligned} \begin{aligned} {\hat{a}}^{\alpha ,\beta }_i&=\frac{(\beta +1+2i+\alpha )(\beta +2+2i+\alpha )}{(i+1)2(1+i+\beta +\alpha )},\\ {\hat{b}}^{\alpha ,\beta }_i&=-\frac{(2i+\beta +\alpha +1)({\alpha }^2-{\beta }^2)}{(1+i)2(i+\beta +\alpha +1)(\beta +2i+\alpha +)},\\ {\hat{c}}^{\alpha ,\beta }_i&=\frac{(2i+\beta +\alpha +2)(i+\alpha )(i+\beta )}{(1+i)(i+\beta +\alpha +1)(2i+\beta +\alpha )}. \end{aligned} \end{aligned}$$

The Legendre polynomial $L_i (x)$ is a special case of the Jacobi polynomial, namely

24 $$\begin{aligned} \begin{aligned} L_i (x) = J^{0 ,0 }_{i}(x) = {}_2F_1 \left( { - i,1+i ;1;-\frac{{x-1 }}{2}} \right) . \end{aligned} \end{aligned}$$

The weight function that ensures the orthogonality of Jacobi polynomials is given by $\omega ^{\alpha ,\beta }(x)=(1+x)^\beta (1-x)^\alpha$, namely,

25 $$\begin{aligned} \int _{-1}^{1} {J_i^{\alpha ,\beta }(x)J_j^{\alpha ,\beta }(x)\omega ^{\alpha ,\beta }(x)dx} = \gamma _i^{\alpha ,\beta } \delta _{ij}, \end{aligned}$$

where $\delta _{ij}$ is the Kronecker Delta symbol, and

26 $$\begin{aligned} \gamma _i^{\alpha ,\beta } = \frac{{2^{(\alpha + \beta +1)} \Gamma (1+i + \beta )\Gamma (1+i + \alpha )}}{{i!( \alpha +2i + \beta + 1)\Gamma ( \alpha + \beta +i + 1)}}. \end{aligned}$$

#### Lemma 2.3

(see for example^37^) *For* $\alpha >0$, *one has*

27 $$\begin{aligned} \begin{aligned} {}_{ - 1}D_{{{\hat{x}}}}^\alpha L_q ({{\hat{x}}})&= \frac{{\Gamma (q + 1)}}{{\Gamma (q - \alpha + 1)}}(1 + {{\hat{x}}})^{ - \alpha } J_q^{\alpha , - \alpha } ({{\hat{x}}}),\quad {{\hat{x}}} \in [ - 1,1],\\ {}_{{{\hat{x}}}}D_1^\alpha L_q ({{\hat{x}}})&= \frac{{\Gamma (q + 1)}}{{\Gamma (q - \alpha + 1)}}(1 - {{\hat{x}}})^{ - \alpha } J_q^{ - \alpha ,\alpha } ({{\hat{x}}}),\quad {{\hat{x}}} \in [ - 1,1]. \end{aligned} \end{aligned}$$

We introduce the following rescale functions:

$$ \begin{array}{*{20}l}    { \wedge :[a,b] \to [ - 1,1]:x \mapsto \frac{{2x - (a + b)}}{{b - a}}} \hfill  \\    { \wedge ^{{ - 1}} :[ - 1,1] \to [a,b]:t \mapsto \frac{{(b - a)t + a + b}}{2}} \hfill  \\   \end{array}  $$

and we write $\wedge (x)$ as ${{\hat{x}}}$. The basis functions selected for the spatial discretization are given by^38,39^:

28 $$\begin{aligned} \varphi _n (x) = L_n ({{\hat{x}}}) - L_{n + 2} ({{\hat{x}}})=\frac{{2n + 3}}{{2(n + 1)}}(1 - {{\hat{x}}}^2 )J_n^{1,1} ({{\hat{x}}}) ,\quad x \in [a,b]. \end{aligned}$$

The approximation space $$V_N^0 $$ can be considered as follows:

29 $$\begin{aligned} V_N^0 =\text {span} \left\{ {\varphi _n (x),\quad n = 0,1,\ldots ,N - 2} \right\} . \end{aligned}$$

The approximate solutions $\psi _N^{j+1}$ and $\phi _N^{j+1}$ are shown as

30 $$\begin{aligned} \psi _N^{j+1} (x) = \sum \limits _{i = 0}^{N - 2} {{\hat{\psi }}_i^{j+1} \varphi _i (x)} ,\qquad \phi _N^{j+1}(x) = \sum \limits _{i = 0}^{N - 2} {{\hat{\phi }}_i^{j+1} \varphi _i (x)} , \end{aligned}$$

where ${\hat{\psi }}_i^{j+1}$ and ${\hat{\phi }}_i^{j+1}$ are the unknown expansion coefficients to be determined. Choosing $v = \varphi _k ,\ 0 \le k \le N - 2$, the representation matrix of the Alikhanov *L*2-$1_{\sigma }$ spectral Legendre–Galerkin numerical scheme has the following representation:

31 $$\begin{array}{*{20}l}\left[ i\,M + \varepsilon \sigma c_{\alpha } \xi _{j}^{\left( {\beta ,\sigma ,\gamma } \right) } \left( {S + S^T } \right) \right] \Psi ^{j + 1} = R_1^j - \sigma \xi _{j}^{\left( {\beta ,\sigma ,\gamma } \right) } H_{1}^{j + 1} , \\ \left[ i\, M + \varepsilon \sigma c_{\alpha } \xi _{j}^{\left( {\beta ,\sigma ,\gamma } \right) } \left( {S + S^T } \right) \right] \Phi ^{j + 1} = R_2^j - \sigma \xi _{j}^{\left( {\beta ,\sigma ,\gamma } \right) } H_{2}^{j + 1} , \end{array}$$

where

32 $$\begin{aligned} \begin{aligned} \Psi ^j&= ({\hat{\psi }}_0^j ,{\hat{\psi }}_1^j ,\ldots ,{{\hat{\psi }}}_{N - 2}^j )^T,\quad \Phi ^j = ({{\hat{\phi }}}_0^j ,{{\hat{\phi }}}_1^j ,\ldots ,{{\hat{\phi }}}_{N - 2}^j )^T,\\ s_{ij}&= \int _\Omega {{}_aD_x^{\frac{\alpha }{2}} \varphi _i (x){}_xD_b^{\frac{\alpha }{2}} \varphi _j (x)dx} , \qquad S = \left( {s_{ij} } \right) _{i,j = 0}^{N - 2} , \\ m_{ij}&= \int _\Omega {\varphi _i (x)\varphi _j (x)dx} ,\qquad \qquad \quad M = \left( {m_{ij} } \right) _{i,j = 0}^{N - 2} , \\ h_{1,i}^j&= \int _\Omega {\varphi _i (x)I_N \left[ {\left( {k_1 \left| {\psi _N^j } \right| ^2 + \left( {k_1 + 2k_2 } \right) \left| {\phi _N^j } \right| ^2 } \right) \psi _N^j + \xi \phi _N^j } \right] dx},\\ h_{2,i}^j&= \int _\Omega {\varphi _i (x)I_N \left[ {\left( {k_1 \left| {\phi _N^j } \right| ^2 + \left( {k_1 + 2k_2 } \right) \left| {\psi _N^j } \right| ^2 } \right) \phi _N^j + \xi \psi _N^j } \right] dx}, \\ H_{1}^j&= (h_{1,0}^j ,h_{1,1}^j ,\ldots ,h_{1,N - 2}^j )^T , \qquad \quad H_{2}^j = (h_{2,0}^j ,h_{2,1}^j ,\ldots ,h_{2,N - 2}^j )^T ,\\ K_1^{j}&=\sum \limits _{i = 0}^{j} {{{\tilde{d}}}_{i}^{(j,\beta ,\sigma )} M\Psi ^i}, \quad \qquad K_2^{j} =\sum \limits _{i = 0}^{j} {{{\tilde{d}}}_{i}^{(j,\beta ,\sigma )} M\Phi ^i},\\ R_1^j&=-\varepsilon \, c_{\alpha }\, (1 - \sigma )\xi _{j}^{\left( {\beta ,\sigma ,\gamma } \right) } \left( {S + S^T } \right) \Psi ^j - (1 - \sigma )\xi _{j}^{\left( {\beta ,\sigma ,\gamma } \right) } H_{1}^j - K_1^j ,\\ R_2^j&=-c_{\alpha } \varepsilon \, (1 - \sigma )\xi _{j}^{\left( {\beta ,\sigma ,\gamma } \right) } \left( {S + S^T } \right) \Phi ^j - (1 - \sigma )\xi _{j,2}^{\left( {\beta ,\sigma ,\gamma } \right) } H_{2}^j - K_2^j . \end{aligned} \end{aligned}$$

#### Lemma 2.4

[see^36,38^] *The elements of the stiffness matrix* *S* *in* (31) *are given by*

33 $$\begin{aligned} s_{ij} = a_i^j - a_i^{j + 2} - a_{i + 2}^j + a_{i + 2}^{j + 2}, \end{aligned}$$

*where*

34 $$\begin{aligned} \begin{aligned} a_i^j&= \int _\Omega {{}_aD_x^{\frac{\alpha }{2}} L_i ({{\hat{x}}}){}_xD_b^{\frac{\alpha }{2}} L_j ({{\hat{x}}})dx}\\&=\left( {\frac{{b - a}}{2}} \right) ^{1 - \alpha } \frac{{\Gamma (i + 1)\Gamma (j + 1)}}{{\Gamma (i - \frac{\alpha }{2} + 1)\Gamma (j - \frac{\alpha }{2} + 1)}}\sum \limits _{r = 0}^N {\varpi _r^{ - \frac{\alpha }{2}, - \frac{\alpha }{2}} J_i^{\frac{\alpha }{2}, - \frac{\alpha }{2}} \left( x_r^{ - \frac{\alpha }{2}, - \frac{\alpha }{2}} \right) J_j^{ - \frac{\alpha }{2},\frac{\alpha }{2}} \left( x_r^{ - \frac{\alpha }{2}, - \frac{\alpha }{2}} \right) }, \end{aligned} \end{aligned}$$

*and* $\left\{ {x _r^{ - \frac{\alpha }{2}, - \frac{\alpha }{2}} ,\varpi _r^{ - \frac{\alpha }{2}, - \frac{\alpha }{2}} } \right\} _{r = 0}^N$ *are the Gauss-Jacobi points and their weights corresponding to the weight function* $\omega ^{ - \frac{\alpha }{2}, - \frac{\alpha }{2}}.$ *The mass matrix* *M* *is symmetric and its nonzero elements are given as*

35 $$\begin{aligned} \begin{aligned} m_{ij} = m_{ji} = \left\{ \begin{array}{l} \frac{b-a}{{2j + 1}} + \frac{b-a}{{2j + 5}},\quad i = j, \\ \\ - \frac{b-a}{{2j + 5}},\quad \quad i = j + 2. \\ \end{array} \right. \end{aligned} \end{aligned}$$

Also, as $H_{p}^{j+1,r} = H_{p}^{j+1 } (\psi _N^{j+1,r},\phi _N^{j+1,r} ),$ $p=1,2$, $r\ge 0,$ the linear system (31) can be solved by Algorithm 1.



## Results and discussions

In this section, we conduct numerical simulations to investigate the modeling capability of the space-time fractional Schrödinger equation (36). Computationally, we consider $N=150$ and $M=1500.$

### Example 1

We consider the following weakly coupled system:

36 $$\begin{aligned} \left\{ \begin{array}{l} i\frac{{\partial ^\beta \psi }}{{\partial t^\beta }} - \left( { - \Delta } \right) ^{\alpha /2} \psi + \left[ { \left| \psi \right| ^2 + \left| \phi \right| ^2 } \right] \psi = 0,\\ \\ i\frac{{\partial ^\beta \phi }}{{\partial t^\beta }} - \left( { - \Delta } \right) ^{\alpha /2} \phi + \left[ { \left| \phi \right| ^2 + \left| \psi \right| ^2 } \right] \phi = 0,\\ \end{array} \right. \end{aligned}$$

with the initial conditions

37 $$\begin{aligned} \begin{aligned} \psi (x,0)&= p_1 \text {sech}\left( {p_1 x + D} \right) e^{iH_0 x} ,\\ \phi (x,0)&= p_2 \text {sech}\left( {p_2 x-D} \right) e^{-iH_0 x} , \end{aligned} \end{aligned}$$

where $$p_1=p_2=1$$, $$D=5$$ and $$H_0=3$$.

In the integer order case, i.e. for $\alpha =2$ and $\beta =1$, the wave propagates from the left to the right spatial domain with a fixed angle. The model is the Manakov system. The collision is elastic and the system is completely integrable, the two waves cross each other, and their velocity and shape are unchanged. It is well known that the standard integer-order Schrödinger equation generates very diffusive numerical solutions. This can be observed here when $\alpha$ is close to 2 and $\beta$ is close to 1.

**Figure 1:**
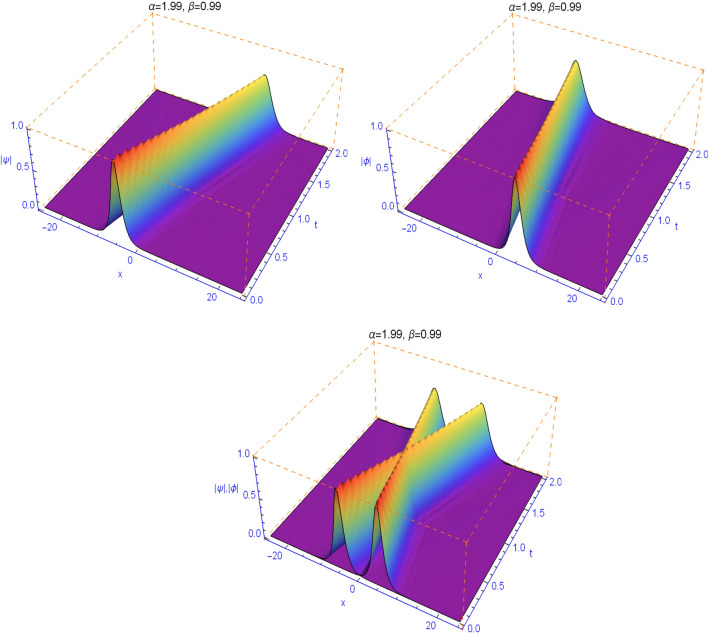
Plots of model (36) for $\beta =0.99$ and $\alpha =1.99$.

**Figure 2:**
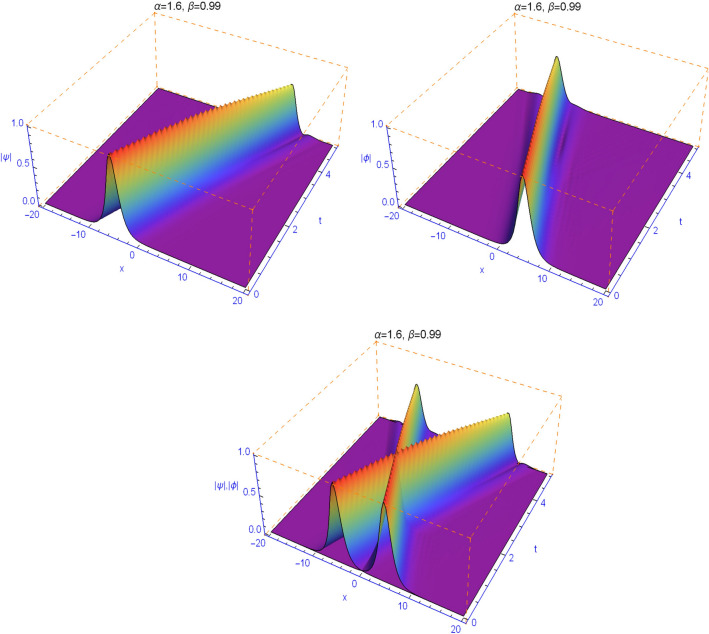
Plots of model (36) for $\beta =0.99$ and $\alpha =1.6$.

**Figure 3:**
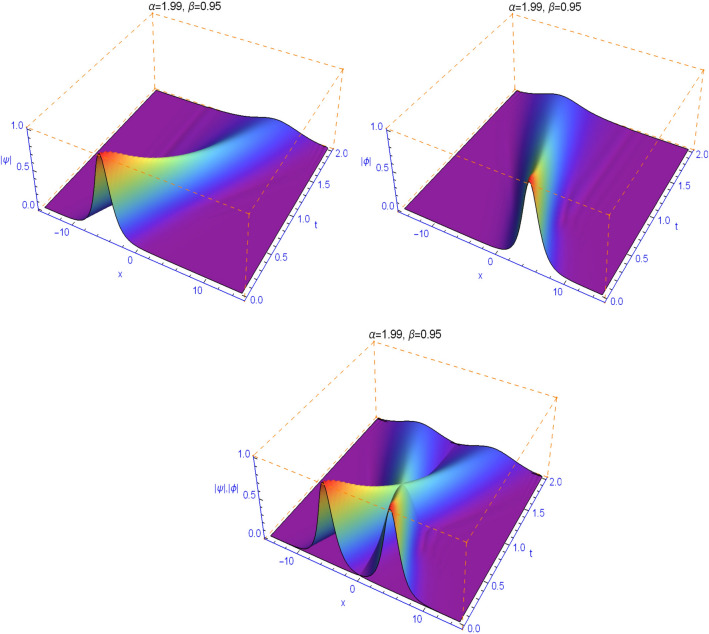
Plots of model (36) for $\beta =0.95$ and $\alpha =1.99$.

**Figure 4:**
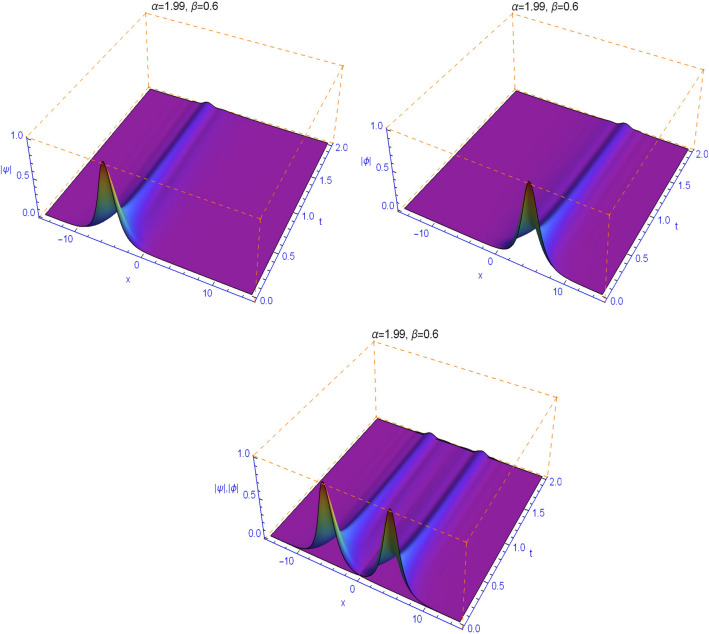
Plots of model (36) for $\beta =0.6$ and $\alpha =1.99$.

**Figure 5:**
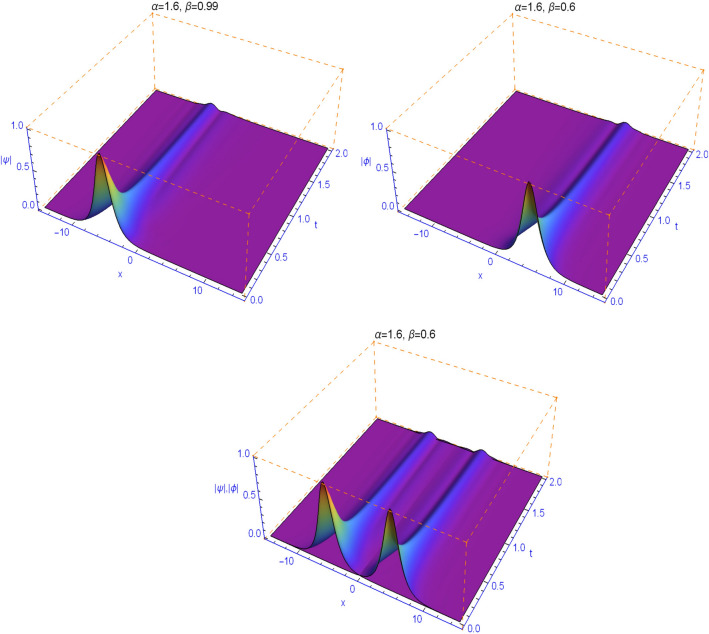
Plots of model (36) for $\beta =0.6$ and $\alpha =1.6$.

The numerical results in Figs. 1, 2, 3, 4 and 5 display the effect of the Lévy index $1<\alpha \le 2$ and the temporal fractality $0<\beta \le 1$ on the shapes and stability of the soliton solutions. These results represent the performance of the space-time fractional Schrödinger equation (35). We find that when $\alpha \ne 2$ and $\beta \ne 1$, the collision is not elastic. The non-integer orders significantly affect the shape of the solitons. One can see in Figs. 1 and 2 that, as the value of the space fractional-order $\alpha$ is decreased, the shape of the solitons changes more quickly.

**Figure 6:**
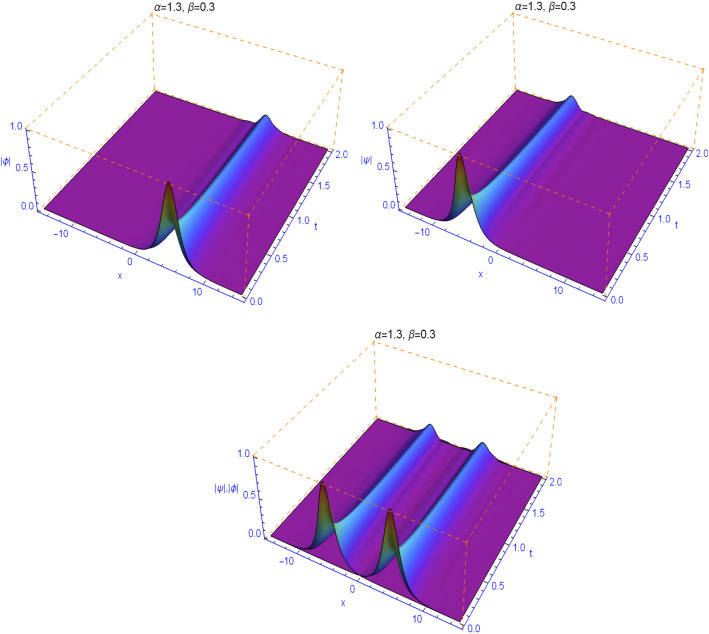
Plots of model (36) for $\beta =0.3$ and $\alpha =1.3$.

From Figs. 3, 4, 5 and 6, we note that the distinct selections of the fractional-order parameters $\beta$ yield simulation results with different decay time or decay properties in the time direction. We observe that as the value of the time fractional-order $\beta$ is decreased, the interface grows sharper and sharper at the beginning of time where-after the solution propagates straight. Here, we notice a new phenomenon that as $\beta$ is decreased, the decay behavior is gradually reduced too. The situation tends to the conventional case when $\beta$ approaches 1. These features can be employed in physics to tunable sharpness of the space-time fractional Schrödinger equation (36) with the different choices of the space fractional order $\alpha$ and the time fractional order $\beta$, without changing the nonlinearity and dispersion effects.

### Example 2

We consider the following strongly coupled system:

38 $$\begin{aligned} \left\{ \begin{array}{l} i\frac{{\partial ^\beta \psi }}{{\partial t^\beta }} - \left( { - \Delta } \right) ^{\alpha /2} \psi + \left[ { \left| \psi \right| ^2 + \left| \phi \right| ^2 } \right] \psi + \gamma \phi = 0,\quad x \in (-25,25) ,\ t \in (0,40],\\ \\ i\frac{{\partial ^\beta \phi }}{{\partial t^\beta }} - \left( { - \Delta } \right) ^{\alpha /2} \phi + \left[ { \left| \phi \right| ^2 + \left| \psi \right| ^2 } \right] \phi + \gamma \psi = 0,\quad x \in (-25,25) ,\ t \in (0,40],\\ \end{array} \right. \end{aligned}$$

subject to the initial conditions

39 $$\begin{aligned} \begin{aligned} \psi (x,0)&= \sqrt{2} \text {sech}\left( { x + \frac{D}{2}} \right) e^{i\frac{{H_0 }}{4}x} ,\\ \phi (x,0)&= \sqrt{2} \text {sech}\left( { x - \frac{D}{2}} \right) e^{-i\frac{{H_0 }}{4}x} , \end{aligned} \end{aligned}$$

where $$D=25$$ and $$H_0=1$$.

**Figure 7:**
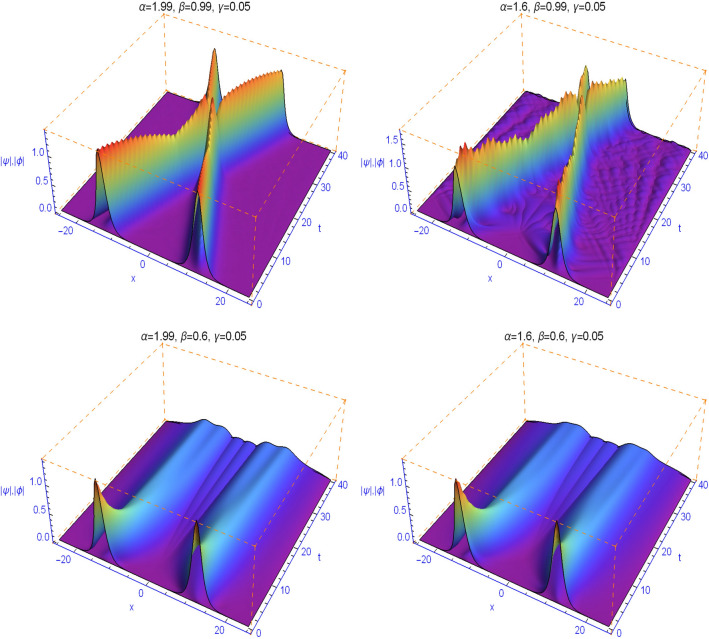
Numerical simulations for Example 2 with $\gamma =0.05$ and different values of the space and time fractional orders.

**Figure 8:**
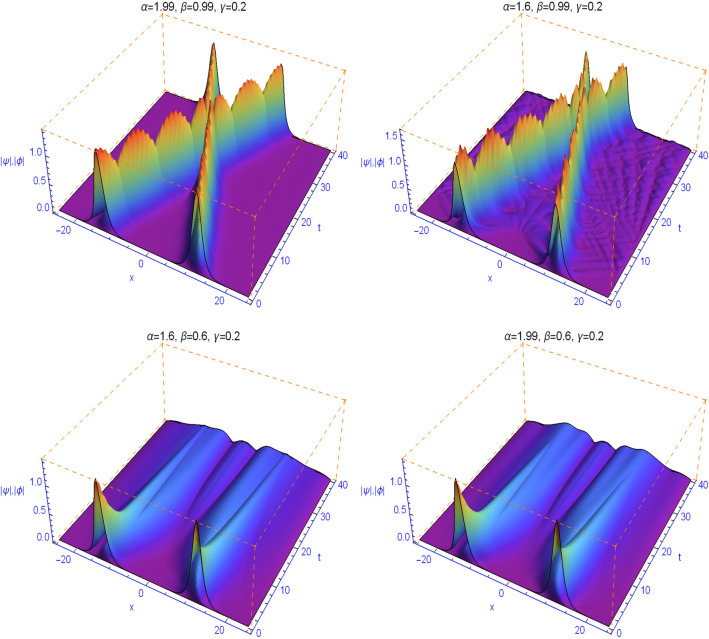
Numerical simulations for Example 2 with $\gamma =0.2$ and different values of the space and time fractional orders.

It is well known that the linear coupling parameter $\gamma$ dramatically affects the collision of solitary waves^40,41^. In Figs. 7 and 8 we study the effect of the linear coupling parameter $\gamma$, the spatial fractional diffraction order $1<\alpha \le 2$ and the temporal fractality $0<\beta \le 1$ on the collision of solitary waves. We find when $\alpha$ tends to 2 and $\beta$ tends to 1 that as the linear coupling parameter $\gamma$ is increased, the jump behavior gets stronger. Moreover, for any $1<\alpha \le 2$ and when $\beta$ tends to 1, the collision is always elastic. The collision will occur earlier with increasing $\alpha$ and become closer to the classical integer-order case. For the space fractional Schrödinger equation with $\beta =1$, the two soliton waves collide and keep their shapes when moving away after the collision. This can be observed here when $\beta$ tends to 1. For smaller values of $\beta$, this feature is absent, the waves do not even cross, and the evolution of the solution is pretty sharp during the first time steps. We note also that the distinct selections of the fractional-order parameters $\beta$ yield simulation results with decay properties in the time direction, see Figs. 7 and 8 (second row). At the same time, some waves are created with oscillations corresponding to fluctuations and indicating the presence of decoherence phenomena that depends on $\beta$.

## Conclusion

In this paper, we numerically investigated the nontrivial behavior of wave packets in a new type of time-space fractional quantum coupler. The model involving a coupled system of time and space fractional nonlinear Schrödinger equations with linear or nonlinear coupling, can be used to uncover a wealth of information about an extended variety of phenomena, such as modeling the Bose-Einstein condensates, the interaction between pulses in nonlinear optics, or signals in nonlinear acoustic media. The time-fractional derivative was considered to describe the nonlocal memory or decay behavior of the model. The numerical simulation for modelling such kind of quantum couplers was carried using an easy to implement algorithm based on a novel consistent scheme. It is a combination of Galerkin spectral method of Legendre type, to approximate the spatial operators, and a high order finite difference method, to approximate the temporal derivatives. The effect of fractality parameters $\alpha$ and $\beta$ on the behaviour of the solution is discussed. The numerical simulations of the time-space fractional nonlinear Schrödinger equations in this paper show that the problem displays time decay behavior that has not been observed before in the time-fractional partial differential equations modeling. Hence, the time and space fractional order operators can be used to control the decay behavior or the memory and the sharpness of the interface and undergo a seamless transition of the fractional order parameters. Of particular interest for this work also is the linear coupling parameter $\xi$. We test the effect of this parameter on the collision of solitary waves. These features can be employed in physics to tunable sharpness of the space-time fractional Schrödinger equation with the different choices of the space fractional order $\alpha$ and the time fractional order $\beta$, without changing the nonlinearity and dispersion effects.
